# Affective Computing for Late-Life Mood and Cognitive Disorders

**DOI:** 10.3389/fpsyt.2021.782183

**Published:** 2021-12-23

**Authors:** Erin Smith, Eric A. Storch, Ipsit Vahia, Stephen T. C. Wong, Helen Lavretsky, Jeffrey L. Cummings, Harris A. Eyre

**Affiliations:** ^1^The PRODEO Institute, San Francisco, CA, United States; ^2^Organisation for Economic Co-operation and Development (OECD), Paris, France; ^3^Department of Neurology & Neurological Sciences, Stanford University, Stanford, CA, United States; ^4^Global Brain Health Institute, University of California, San Francisco, San Francisco, CA, United States; ^5^Global Brain Health Institute, Trinity College Dublin, Dublin, Ireland; ^6^Department of Psychiatry and Behavioral Sciences, Baylor College of Medicine, Houston, TX, United States; ^7^Division of Geriatric Psychiatry, McLean Hospital, Boston, MA, United States; ^8^Division of Geriatric Psychiatry, Harvard Medical School, Boston, MA, United States; ^9^Systems Medicine and Biomedical Engineering Houston Methodist, Houston, TX, United States; ^10^Semel Institute for Neuroscience and Human Behavior, University of California, Los Angeles, Los Angeles, CA, United States; ^11^Chambers-Grundy Center for Transformative Neuroscience, Department of Brain Health, School of Integrated Health Sciences, University of Nevada, Las Vegas (UNLV), Las Vegas, NV, United States; ^12^IMPACT, The Institute for Mental and Physical Health and Clinical Translation, Deakin University, Geelong, VIC, Australia

**Keywords:** affective computing, late-life depression, dementia, Alzheimer's disease, digital phenotyping

## Abstract

Affective computing (also referred to as artificial emotion intelligence or emotion AI) is the study and development of systems and devices that can recognize, interpret, process, and simulate emotion or other affective phenomena. With the rapid growth in the aging population around the world, affective computing has immense potential to benefit the treatment and care of late-life mood and cognitive disorders. For late-life depression, affective computing ranging from vocal biomarkers to facial expressions to social media behavioral analysis can be used to address inadequacies of current screening and diagnostic approaches, mitigate loneliness and isolation, provide more personalized treatment approaches, and detect risk of suicide. Similarly, for Alzheimer's disease, eye movement analysis, vocal biomarkers, and driving and behavior can provide objective biomarkers for early identification and monitoring, allow more comprehensive understanding of daily life and disease fluctuations, and facilitate an understanding of behavioral and psychological symptoms such as agitation. To optimize the utility of affective computing while mitigating potential risks and ensure responsible development, ethical development of affective computing applications for late-life mood and cognitive disorders is needed.

## Introduction

Between 2019 and 2050, the number of people aged 65 years or over in the world will increase from 703 million to 1.5 billion people (1). With the rapid growth in the aging population around the world, improving the standard of care in late-life mood and cognitive conditions is of the utmost importance. Late-life mood and cognitive conditions are characterized by their complexity, multisystemic nature and broad societal impact, hence making them poorly suited to siloed approaches of thinking and innovation (2). Issues such as overlapping symptoms, comorbidities, and misdiagnosis among mental health and neurological disorders represents only a small subset of the challenges facing late-life mood and cognitive conditions (2). For example, psychiatric symptoms often occur during prodromal stages of neurodegenerative diseases (3–5). Neurodegenerative diseases are often misclassified as psychiatric disease, which can cause the patient to receive delayed, inappropriate treatment and experience more distress (4). Relatedly, many neurodegenerative conditions have clinical and neuropathological overlap, which can cause difficulty with accurate diagnosis and treatment (6). Comorbidities also present further challenges. As an example, 61% of people with dementia are estimated to have three or more comorbid diagnoses (7), which often remain over or under-treated and negatively affect the physical and psychological well-being of people with dementia (8). As the burden of late-life mood and cognitive disorders continues to rise, improved prevention, diagnosis, and treatment options are urgently needed (9, 10). To solve the unprecedented complexities and challenges associated with late-life mood and cognitive conditions, new technologies and approaches to care are needed. Affective computing has immense potential to benefit the treatment and care of late-life mood and cognitive disorders. Affective computing (also referred to as artificial emotion intelligence or emotion AI) is the study and development of systems and devices that can recognize, interpret, process, and simulate emotion or other affective phenomena (11, 12). It is a transdisciplinary field that combines engineering and computer science with psychology, cognitive science, neuroscience, sociology, education, psychophysiology, value-centered design, ethics, and more.

In this paper, we aim to provide an overview of common affective computing modalities with applications for late-life mood and cognitive disorders. We additionally explore specific applications for late-life depression and Alzheimer's disease (AD). Lastly, we discuss ethical implications and analyze key challenges that must be overcome to ensure ethical development of affective computing for late-life mood and cognitive conditions.

## Methods

Publications were collected in September 2021 from four databases: PubMed, PsycINFO, OvidSP, and Web of Science. To construct the search protocol, the research question was structured in terms of the following topics: Affective Computing, digital phenotyping, late-life depression, dementia, Alzheimer's disease, and ethical issues. Synonyms and main terms for these topics were selected to construct the search codes. After an initial search, publication titles and abstracts were screened according to year of publication, publication in English language, and of peer-reviewed type. A total of 150 articles were utilized in this review.

## Results

### Affective Computing and Late-Life Depression

Depression is the leading cause of disability worldwide and a major contributor to the overall global burden of disease (13). The scope and burden of late-life depression is significant and expected to rise in the 21^st^ century (14). Depression is estimated to affect 29% of elderly Europeans (15) and 30.6% of elderly Chinese (16). When compared to younger patients with depression, older adults with depression typically have more medical and neurologic comorbidities and display more cognitive impairment (17). Data suggest that 1 in 10 cases of dementia world-wide can be attributed to depression (18). Neurotoxicity due amyloid and tau protein aggregation may represent a pathophysiological cascade which, along with vascular compromise, may predispose individuals to late-life depression (17). Table 1 highlights clinical challenges in late-life depression and ways in which affective computing may be beneficial.

**Table 1:** Affective computing applications for clinical challenges in late-life depression.

	**Vocal biomarkers**	**Facial expression biomarkers**	**Body movements**	**Eye movements**	**Keystroke dynamics**	**Social media behavior**	**Socially assistive robots (SARs)**
**APPROACHES FOR AFFECTIVE COMPUTING IN CLINICAL CHALLENGES OF LATE-LIFE DEPRESSION**							
Inadequacies of current screening and diagnostic approaches	Use vocal biomarkers to detect depression (19–23)	Use facial expression biomarkers to detect depression (23–30)	Use head movements and pose to detect depression (26, 30–32)	Use gaze and eye movement to detect depression to detect behavioral consistent with depression (33)	Use keystroke dynamics to detect typing behavior associated with depression (34)	Use behavioral attributes related to social engagement, emotion, language, linguistic style, and writing aspects to detect depression (35–40)	Use SARs to administer screening and diagnostic approaches that leverage affective computing biomarkers (41)
Trial-and-error treatment approaches	Monitor depression severity and treatment response to determine optimal treatment using vocal biomarkers (42–45)	Monitor depression severity and treatment response to determine optimal treatment using facial expression biomarkers (46)	Leverage body and head movement analysis to measure depression severity throughout the course of treatment to determine optimal approach (46, 47)	Measure depression treatment response using anti-saccade eye movement tasks (48)	Determine optimal treatment by measuring depression severity via touchscreen typing (49)	Assess social media behavior and develop treatment strategy for social media usage that reinforces depressive beliefs and symptoms to improve overall treatment outcomes (50)	Use SARs to provide in-home therapeutic approach and collect real-time data on affective computing biomarkers to determine optimal treatment strategies (41)
Loneliness and social isolation	Detect loneliness and social isolation and better identify behavioral phenotypes of loneliness and social isolation through vocal biomarkers (51)	Assess spontaneous smile mimicry to detect and monitor loneliness (52)	Assess body movement coordination, which may be impaired during loneliness due to changes in the left posterior superior temporal sulcus (53)	Assess eye movement, which may be impaired during loneliness due to changes in the left posterior superior temporal sulcus (53)	Assess keystroke dynamics and hand action, which may be impaired during loneliness due to changes in the left posterior superior temporal sulcus (53)	Monitor social media behavior for early detection of loneliness (54)	Use socially assistive robots (SARs) or social companion robots that have affective computing capabilities to help older adults with depression (55–57)
Poor treatment follow-up	Monitor daily fluctuations using vocal biomarkers during time outside of the clinic and receive alerts if problems with treatment (19, 23)	Monitor daily fluctuations using facial expression biomarkers during time outside of the clinic and receive alerts if problems with treatment (23)	Assess body movement during time outside of the clinic to better understand symptom fluctuations (46, 47)	Track eye movement to understand depression symptoms between in-clinic visits (48)	Monitor keystroke behavior to assess treatment efficacy (34)	Monitor behavioral attributes related to social engagement, emotion, language, linguistic style, and writing aspects to monitor depression symptom severity between treatment sessions or clinic visits (35–40)	Use SARs to interact with older adults and understand depression symptom progression and severity (55–57)
Co-occurrence with anxiety disorders	Differentiate between depression and anxiety disorders using vocal biomarkers	Differentiate between depression and anxiety disorders using facial expression biomarkers	Identify and monitor anxiety and depression severity scores using digital gait movement (58)	Assess anxiety and depression severity using eye movement (59)	Monitor and differentiate between anxiety and depression via touchscreen typing (34)	Detect anxiety and depression via social media behavior (40)	Capture affective computing biomarkers to differentiate between anxiety and depression via SARs (41)
Co-occurrence with Alzheimer's disease	Monitor symptoms associated with depression and Alzheimer's disease using vocal biomarkers (60)	Monitor symptoms associated with depression and Alzheimer's disease using facial expression biomarkers (61)	Kinematic analysis can detect co-morbid Alzheimer's disease for patients with depression (62)	Detect and differentiate between Alzheimer's and depression via eye movement tracking (63, 64)	Monitor early stages of Alzheimer's disease and depression via touchscreen typing (34, 65)	Detect late-life depression in Alzheimer's disease patients via speech and language analysis on social media (66)	Use SARs to analyze speech and language of people with late-life depression and Alzheimer's disease (66)
Risk of suicide	Detect suicidal ideation and risk using vocal biomarkers (67)	Evaluate risk of suicide using facial expression biomarkers (68)	Monitor body movement to detect risk of suicide (69)	Assess eye movement to identify attention bias for suicide related stimuli (70)	Use digital phenotyping from smartphone typing to detect risk of suicide (69)	Detect suicidal ideation and risk using social media behavioral analysis (71, 72)	For people at risk of suicide, SARs can be leveraged to track affective computing biomarkers that indicate risk of suicide (69)

Williamson et al. (23) examined changes in motor output in people with depression from vocal acoustics and facial movements (23). Using the 4th International Audio/Video Emotion Challenge (AVEC), which consists of a read passage and free-response speech segment from subjects with varying depression levels according to their self-reported Beck depression inventory assessment, they developed a multimodal analysis pipeline that leverages complementary information in audio and video signals including structure and timing features for estimating depression severity. Using the identified features of changes in coordination, movement, and timing of vocal and facial movements, the developed algorithm was able to predict the Beck depression inventory ratings from the AVEC test set with a root-mean-square error of 8.12 and mean absolute error of 6.31 (23).

De Choudhury et al. (36) used behavioral attributes from social media to characterize severity of depression at a population level by developing a social media depression index (SMDI) (73). Using crowdsourcing techniques, they built a corpus of over 69K Twitter postings shared by individuals diagnosed with clinical depression that was measured using the Center for Epidemiologic Studies Depression Scale (CES-D) screening test. By analyzing behavioral features including emotional expression, linguistic style, user engagement, and egocentric social network properties, they built a model that can predict if a post is indicative of depression with an accuracy of more than 70% and precision of 0.82. Lastly, they developed the SMDI metric. The SMDI metric leverages the prediction model to predict posts indicative of depression on Twitter and helps characterize the levels of depression in populations. The geographical, demographic, and seasonal patterns of depression given by SMDI confirm known clinical characteristics of depression and are highly correlated with depression statistics reported by the Centers for Disease Control and Prevention (CDC) (73).

Mundt et al. (42) used vocal acoustic biomarkers to predict depression severity and treatment response (42). One hundred five adults with depression were recruited into a 4-week, randomized, double-blind, place-controlled clinical trial. Speech samples were collected at baseline and study end point using an automated telephone system. Clinician-rated and patient-reported measures of depression severity and treatment response were collected. Results from the study replicated and supported findings from prior studies. More severe depression produced longer recordings with more pause time, more variable pause lengths, a greater percentage of pause time, smaller speech/pause ratios, and slower speaking rates. Speech pause times were found to shorten with clinical improvement following treatment, and depressed patients who did not improve clinically were found to have smaller vocal acoustic changes and/or changes that were directionally opposite to treatment responders.

### Affective Computing and Alzheimer's Disease

Alzheimer's disease (AD) is the most common neurodegenerative disorder and largest cause of dementia in the world with rapidly growing personal, societal, economic, and medical implications. In the United States alone, over 6.2 million people suffer from AD that costs the healthcare system more than $355 billion annually, not including the value of informal caregiving (74). At a global level, there are more than 35 million people currently living with AD, and by 2050 the number is expected to more than triple, exceeding 115 million people (75). There is growing interest in detecting AD during prodromal stages because (a) the likelihood of reversing anatomic and physiologic changes (such as neuronal death) likely decreased dramatically as the disease advances (61, 76), (b) there is a growing body of evidence that cognitive, sensory, and motor changes may precede clinical manifestation of AD by 10–20 years (74, 76), and (c) aducanumab, the only approved disease-modifying therapy is recommended only for patients with early AD (77). Table 2 highlights clinical challenges in AD and ways in which affective computing may be beneficial.

**Table 2:** Affective computing applications for clinical challenges in AD.

	**Vocal biomarkers**	**Facial expression biomarkers**	**Body movements**	**Eye movements**	**Keystroke dynamics**
**APPROACHES FOR AFFECTIVE COMPUTING IN CLINICAL CHALLENGES OF ALZHEIMER'S DISEASE**					
Lack of early, objective screening and diagnostic approaches	Use vocal biomarkers to detect and access mild cognitive impairment (MCI) and prodromal stages of AD (61, 78–83)	Assess cognitive and neuropsychiatric symptoms of AD using facial expression impairments (84, 85)	Capture motor impairments that precede signs of cognitive impairment by over a decade in people with AD through measuring gait speed, stride length, and gait symmetry (61, 86–90)	Use eye movement to detect MCI and prodromal stages of AD (91–95)	Capture differences in reaction speed and movement that have been found in early stages of AD using an active finger tapping test or passive data collection from daily computer, tablet, or smartphone keyboard use (61, 96–98)
Lack of objective biomarkers for monitoring disease progression and comprehensive, daily fluctuations	Use vocal biomarkers to monitor disease progression (83)	Monitor diseases progression and daily fluctuations of symptoms using facial expressions (84, 85)	Assess gait and balance throughout AD to monitor disease progression (99–101)	Monitor eye movement to track AD progression (102, 103)	Monitor progression of cognitive impairment including MCI to AD using touchscreen typing (61, 65)
Understanding and addressing the behavioral and psychological symptoms, such as agitation and pain, experienced by patients with AD	Capture vocal biomarkers using sensing technology to monitor behavioral and psychological symptoms of AD (104)	Digitize facial expressions and movements to monitor behavioral and psychological symptoms of AD using sensing technology (104)	Use body movements, such as number of transitions between spaces, to detect and better understand different behavioral and psychological symptoms of AD (105)	Use eye movements to understand and monitor behavioral and psychological symptoms of AD via sensing technology (104)	Analyze typing to identify subtypes of AD based on the presence and intensity of behavioral and psychological symptoms of AD
Co-occurrence with depression	Monitor symptoms associated with depression and AD using vocal biomarkers (60)	Monitor symptoms associated with depression and AD using facial expression biomarkers (61)	Kinematic analysis can detect co-morbid AD for patients with depression (62)	Detect and differentiate between AD and depression via eye movement tracking (63, 64)	Monitor early stages of AD and depression via touchscreen typing (34, 65)
Misdiagnosis between AD and other neurodegenerative disorders during early stages of disease progression	Differentiate between AD, Parkinson's disease (AD), and Lewy Body Disease (LBDs) using vocal biomarkers (60, 106–109)	Differentiate between AD and PD using facial expression analysis	Differentiate between AD and PD using digital gait analysis (99–101, 110)	Assess PD, AD, and Lewy body dementia via eye movement analysis (111)	Detect and differentiate early stages of PD and AD using typing and keyboard dynamics (61, 65, 112)

Ahmed et al. (83) examined connected speech as a marker of disease progression in autopsy-proven AD (83). Samples of connected speech were obtained from 15 patients who were part of a longitudinal cohort study in whom AD was diagnosed during life and later confirmed at post-mortem. The study analyzed spoken discourse over the course from MCI to mild AD dementia to moderate AD dementia. Samples were analyzed using measures of syntactic complexity, lexical content, speech production, fluency, and semantic content. Subtle changes were found in spoken language that were detectable in MCI stages and enabled monitoring progression through successive clinical stages of AD. Language biomarkers could help identify prodromal AD and provide a way to monitor disease in therapeutic trials (83).

Bayat et al. (113) evaluated the ability of in-vehicle GPS data loggers and driving behavior to distinguish cognitively normal older drivers with preclinical AD from those without preclinical AD using machine learning algorithms (113). For 1 year, 139 subjects (64 with preclinical AD; 75 without preclinical AD, as determined by cerebrospinal fluid biomarkers) were monitored while they drove with a commercial in-vehicle GPS data logger. Random Forest models were trained on the GPS data. The receiver operating curve (ROC) area under the curve (AUC) for predicting preclinical AD from driving features alone was 0.82, with the addition of age alone increased to 0.94, and with the additions of age and APOE ε4 status increased to 0.96 (113).

Gills et al. (95) developed and validated a short digital eye-tracking assessment that predicts cognitive status among adults (95). Fifty-five adults (11 with MCI and 44 cognitively normal) were tested on two occasions. During the first visit, participants underwent a brief eye-tracking based visual paired-comparison (VPC), Montreal Cognitive Assessment (MoCA), Digital Symbol Coding test (DSC), and NIH Toolbox Cognitive Battery (NIHTB-CB). During the second visit, participants underwent VPC, DSC, NIHTB-CB, and dual-task (DT). VPC reliably predicted cognitive status while demonstrating high test-retest reliability and displayed significant associations with gold standard cognitive assessments (95). Eye-tracking based VPC may provide a useful, brief, and scalable screening tool for cognitive impairment (95).

### Ethical Implications of Affective Computing in Healthcare

New approaches are needed to address the technical, scientific, philosophical, and ethical challenges associated with affective computing applications in healthcare (114). One key challenge is ensuring that the tools account for sex, gender, racial, ethnic, and culture-based differences. For example, two-thirds of AD patients worldwide are women (115, 116). Women have a higher lifetime risk of stroke than men, and women are twice as likely to be diagnosed with depression and anxiety disorders, and migraines (116–118). These are all specific risks factors for developing dementia.

Given the higher rate of depression and AD among women research is needed to understand if and how sex and gender-based differences affect disease manifestations that may result in the need for different digital biomarkers and machine learning affective computing approaches for males vs. females. It is critical to ensure that the machine learning algorithms capture symptoms that may be more common or different in females.

We must ensure that algorithmic fairness with affective computing does not stop at merely accounting for sex and gender-based differences. Data sets and algorithms used for affective computing must also include bias reduction measures that account for ethnic, racial, geographical, cultural, and other human biases (119). As one example, facial analysis algorithms are often trained on datasets that are predominately comprised of lighter-skinned males and may fail to detect female faces and people of different races and ethnicities (120). Algorithmic and human bias must be addressed to ensure greater fairness, transparency, and accountability in the development of affective computing applications.

The various ways affective computing can be leveraged in healthcare also have different ethical implications. For example, current affective computing technologies typically leverage passive or active data collection. In the context of affective computing for healthcare, passive data collection may entail information continuously collected from smartphone usage, driving, or social media, whereas active data collection may involve specific vocal or facial expression assessments within a clinical setting. Different ethical and practical considerations arise for passive vs. active data collection and the setting in which data is conducted (e.g., at home using everyday technological devices or via a specific assessment in-clinic). These challenges must be addressed.

Other ethical considerations that will need to be addressed and mitigated with the advent of affective computing applications in healthcare are (a) addressing privacy and data security concerns; b) determining what it means for people and society to know they have a disorder 10–20 years before clinical manifestations (such as with AD or PD); (c) navigating how to update affective computing models after deployment to make them optimally adaptive and effective; and (d) considering what privacy means when a doctor or friend or family member could tell if you have a certain health condition or are suicidal.

The 2021 “Ethics and governance of artificial intelligence for health: WHO guidance” report by the World Health Organization (WHO) highlights key ethical principles for the use of artificial intelligence in healthcare, which include protecting autonomy; promoting human well-being, human safety and the public interest; ensuring transparency, explainability and intelligibility; fostering responsibility and accountability; ensuring inclusiveness and equity; and promoting artificial intelligence that is responsive and sustainable (121). These ethical principles must guide the development of affective computing applications for late-life mood and cognitive conditions to ensure human rights are upheld and that patient and community interests do not become subordinate to the powerful commercial interest of technology companies or the interests of governments in surveillance and social control (121). Questions related to data security, privacy, and ownership must also be addressed; how CAN these concerns be navigated on a global scale? Initiatives such as the National Institute of Health Bridge to Artificial Intelligence (Bridge2AI) program brings together technologists, biomedical experts, social scientists, and humanists to develop ethical data sets and tools and will help navigate these pressing questions related to affective computing applications in brain health (122).

By addressing the ethical implications and challenges during early stages of technology development, the introduction of affective computing technology in healthcare can bring a new era of health that is marked by a proactive, personalized, and preventative approach to care. Affective computing has the possibility to be applied globally, such as through the ubiquity of smartphones, and can help address challenges related to health equity. Affective computing technology has the potential to improve early detection and screening, disease severity and progression monitoring, treatment efficacy monitoring, and the quality of life for people around the world with a myriad of different neurological health conditions.

## Conclusion

Affective computing can address challenges associated with late-life mood and cognitive conditions, including depression and AD. Affective computing technologies- ranging from vocal dynamics to facial expressions to social media usage to driving behavior- can provide objective biomarkers and tools for early detection, monitoring treatment response, tracking disease progression, and more comprehensively understanding the daily life of patients. To leverage affective computing to increase global brain health equity and a precision medicine approach to care, efforts are needed to ensure ethical development of affective computing for late-life mood and cognitive conditions that account for algorithmic and human bias. With these safeguards affective computing can become a major tool of care of late life affective and cognitive disorders.

## Author Contributions

ES led the manuscript development and wrote the initial review. HE, JC, HL, SW, IV, and EAS contributed edits, additional examples, and ideas. All authors contributed to the idea development, writing, editing of this manuscript, and approved the submitted version.

## Conflict of Interest

The authors declare that the research was conducted in the absence of any commercial or financial relationships that could be construed as a potential conflict of interest.

## Publisher's Note

All claims expressed in this article are solely those of the authors and do not necessarily represent those of their affiliated organizations, or those of the publisher, the editors and the reviewers. Any product that may be evaluated in this article, or claim that may be made by its manufacturer, is not guaranteed or endorsed by the publisher.
